# Device-to-Device (D2D) Multi-Criteria Learning Algorithm Using Secured Sensors

**DOI:** 10.3390/s22062115

**Published:** 2022-03-09

**Authors:** Khalid Haseeb, Amjad Rehman, Tanzila Saba, Saeed Ali Bahaj, Jaime Lloret

**Affiliations:** 1 Department of Computer Science, Islamia College Peshawar, Peshawar 25000, Pakistan; khalid.haseeb@icp.edu.pk; 2 Artificial Intelligence and Data Analytics (AIDA) Lab, CCIS Prince Sultan University, Riyadh 11586, Saudi Arabia or rkamjad@gmail.com (A.R.); or drstanzila@gmail.com (T.S.); 3 MIS Department College of Business Administration, Prince Sattam Bin Abdulaziz University, Alkharj 16278, Saudi Arabia; s.bahaj@psau.edu.sa; 4 Instituto de Investigación para la Gestión Integrada de Zonas Costeras, Universitat Politenica de Valencia, 46379 Gandia, València, Spain; 5 School of Computing and Digital Technologies, Staffordshire University, Stoke ST4 2DE, UK

**Keywords:** wireless systems, mobile sensors, D2D, technological development, Internet of things

## Abstract

Wireless networks and the Internet of things (IoT) have proven rapid growth in the development and management of smart environments. These technologies are applied in numerous research fields, such as security surveillance, Internet of vehicles, medical systems, etc. The sensor technologies and IoT devices are cooperative and allow the collection of unpredictable factors from the observing field. However, the constraint resources of distributed battery-powered sensors decrease the energy efficiency of the IoT network and increase the delay in receiving the network data on users’ devices. It is observed that many solutions are proposed to overcome the energy deficiency in smart applications; though, due to the mobility of the nodes, lots of communication incurs frequent data discontinuity, compromising the data trust. Therefore, this work introduces a D2D multi-criteria learning algorithm for IoT networks using secured sensors, which aims to improve the data exchange without imposing additional costs and data diverting for mobile sensors. Moreover, it reduces the compromising threats in the presence of anonymous devices and increases the trustworthiness of the IoT-enabled communication system with the support of machine learning. The proposed work was tested and analyzed using broad simulation-based experiments and demonstrated the significantly improved performance of the packet delivery ratio by 17%, packet disturbances by 31%, data delay by 22%, energy consumption by 24%, and computational complexity by 37% for realistic network configurations.

## 1. Introduction

IoT-based technologies have gained numerous growth in the development of smart cities and support to real-time communication systems [1,2,3]. Wireless sensor networks (WSN) enable low-power deployments, and they have become the dominating choice in the composition of IoT devices. The technology of WSN is broadly utilized in various applications, such as precision agriculture, healthcare, vehicle transportation, smart cities, etc. [4,5,6]. It is comprised of tiny and battery-powered nodes with limited memory and transmission power, and such constraints restrict the amount of computation in facilitating the network users [7,8,9]. In large network domains, most of the solutions prefer the multi-hop paradigm rather than the single hop, which improves the connectivity among IoT networks and supports the connected devices for transmitting their collected data. However, with increases in data traffic, frequent changes occur in communication channels and most of the network systems degrade their performance in terms of resource management and security. Many solutions have used the techniques of machine learning to make an intelligent decision and forward the monitoring data with nominal overhead [10,11,12]. However, most of the existing solutions are not able to cope with malicious threats in the existence of mobile IoT devices. Accordingly, it is easy to degrade the performance of smart cities while transferring the gathered data among the D2D communication system over the unreliable source of network channels [13,14,15]. Furthermore, due to the constraints in devices for resources, authentication, integrity, and security are other significant parameters in transmitting the smart data from the critical field [16,17,18]. Therefore, network devices must be protected from unauthorized access and maintain their accuracy in terms of privacy and trustworthiness.

This study aims to propose a D2D multi-criteria reinforcement learning algorithm using secured and mobile IoT devices. It offers an efficient way of collecting sensors’ data and utilizes the technique of machine learning with the least overheads on sensors and provides intelligent methods for ensuring low-latency data routing. Additionally, unlike most of the existing solutions, the proposed algorithm increases the robustness of the mobile network for security, given as follows. (i) Mobile devices are authenticated in their transmission radius before connecting to the route discovery process, and after mutual verification, the proposed algorithm allows them to become involved in the data routing. (ii) It offers a trusted analysis of security measurements and improves enhancement in terms of privacy with data integrity in mobile networks. (iii) Moreover, it provides the security flow between three stages i.e., mobile devices, gateways, and the sink node. The significant contributions of the proposed work are as follows:

- D2D authentication algorithm is developed for a mobile network to ensure the authenticity and trustworthiness between partial and fully-connected nodes.
- Using a multi-criteria process, the reinforcement learning technique is applied and the network system is trained using realistic conditions. The proposed algorithm offers the selection of optimal neighbors using the computation of rank value that is comprised of energy, speed, link cost, and radio coverage. Accordingly, it reduces the sizes of routing tables and avoids excessive routing intervals.
- Moreover, the proposed algorithm protects devices and attains uncompromised data against security attacks.
- The simulations are performed to verify the improvement of the proposed algorithm in the comparison of existing work.

The rest of the article is divided into the following subsections: The related work and problem statement are described in Section 2. Section 3 explains the proposed algorithm with flow diagrams. Simulation configuration and experimental results are discussed in Section 4, and Section 5 provides the conclusion with suggestions for future work.

## 2. Related Work

IoT is one of the most promising technologies of the current era and interacts with sensors for observing the physical world [19,20,21]. These technologies have expanded into the real-time environment and support the applications to govern their operations. Recently, many solutions have presented to optimize the transmissions and increase the accuracy of online data retrieval systems. The authors of [22] determined the required resources of energy at the BS for IoT-enabled systems. Numerous agricultural sensors have utilized in precision agriculture for continually monitoring the field and communicating with the smart nodes. They presented a unique product density model for estimating the energy requirements for BS. Additionally, a method for Improved Duty Cycling was provided that makes use of the residual energy parameter. The proposed routing protocol [23] employs a region-based static clustering technique to efficiently cover the agricultural area while utilizing threshold-sensitive hybrid routing to send sensed data to the base station. In addition, the proposed protocol uses fuzzy logic to select the optimal cluster head (CH) among all sensor nodes in a given round, minimizing node energy usage during each data transmission period. The suggested energy-efficient protocol is compared to establish benchmark protocols, such as energy-efficient heterogeneous clustering (EEHC), developed distributed energy-efficient clustering (DDEEC), and region-based hybrid routing (RBHR). The research and testing findings indicate that user-defined transmission thresholds substantially decrease the data transmission rate. Furthermore, the balanced employment of fuzzy logic, static clustering, and hybrid routing effectively reduces the energy consumption of sensor nodes throughout each data transmission round, therefore extending the network’s total lifetime. In [24], the authors proposed PAwCOR to develop a distributed method for the selection of CH by using node energy, latency, and congestion characteristics. Energy saving is accomplished via the use of nodes that are selected depending on sensing inaccuracy. PAwCOR enables the application of periodic data with the least amount of delay possible via the use of various routing routes. Additionally, it fulfills the need for non-delay-tolerant applications by utilizing service differentiation to prioritize time-critical data transmission. By allocating at least one route for both essential and routine data transfers, the suggested method improves performance compared to current protocols. It improved the performance in terms of latency, average energy consumption, packet delivery ratio, and average residual energy to attain reliable transmission. The authors of [25] proposed CTEER, an energy-efficient routing protocol based on cluster trees, to address the fast energy loss experienced by ordinary nodes while using the conventional static routing tree method. This protocol is a rendezvous-based method with a low-delay characteristic. As a result, the protocol is well-suited for time-critical applications, such as network live broadcast systems, automated railway operation systems, ticketing software, and intelligent home systems. It creates a cross-routing tree in which the mobile sink serves as the central node. Clustering algorithms are used to group the ordinary nodes and aggregate the data packets based on the routing tree. The suggested approach outperforms RRP in terms of the network lifecycle, energy consumption, and data latency. The authors of [26] proposed a deep-reinforcement learning-based quality-of-service (QoS)-aware secure routing protocol (DQSP). It aims to ensure the QoS and extract knowledge from traffic history by cooperating with the observing environment. Moreover, the proposed protocol optimizes the policies of routing. It performs significant improvement under different network metrics and has proven high convergence and effectiveness. The authors of [27] presented QL-MAC based on Q-learning, which iteratively tweaks the MAC parameters through a trial-and-error process and attains energy-efficient communication. It offers minimization problems without predetermining the system model, and also provides a self-adaptive protocol in case of topological or any external events. It readjusts the duty cycle of nodes and explicitly minimizes the energy consumption. The large-scale simulation experiments demonstrate its efficacy over other schemes.

It was noticed that technologies of IoT and sensors are performing an extraordinary role in the development of smart communication. The sensors are widely used in different applications, including remote operations to observe the data and respond with a timely reaction [28,29,30]. However, they are bound in terms of resources and limit the online services for IoT networks. Moreover, transporting sensitive data from network devices towards the data centers is another important characteristic for any IoT-enabled system. It has also been seen that different solutions are discussed to improve the energy consumption and QoS parameters by using artificial intelligence and machine learning techniques for D2D communication; however, most of the reinforcement learning solutions lack the optimal consumption of resources, especially in the routing phase for mobile devices. In addition, they are not able to cope with the dynamic evaluation of routing links, and in such cases, sensors’ data were frequently dropped. Moreover, it was also observed that a few solutions are still vulnerable to external attacks and not able to cope with data security under mobile nodes. Such solutions could not provide a robust mutual authentication system, and as a result, communication performance is non-collaborative and uncertain. Table 1 summarizes the discussion of the existing solutions.

## 3. Proposed Multi-Criteria Learning Algorithm Using Secured Sensors

Sensors integrated with IoT objects are utilized in different domains to gather data and support the community using a smart communication system. IoT network provides the processes of data collection and assists the end-users in observing and optimizing the transmission based on environmental conditions. In this section, we present the details of the proposed algorithm and its working flow.

**Figure 1:**
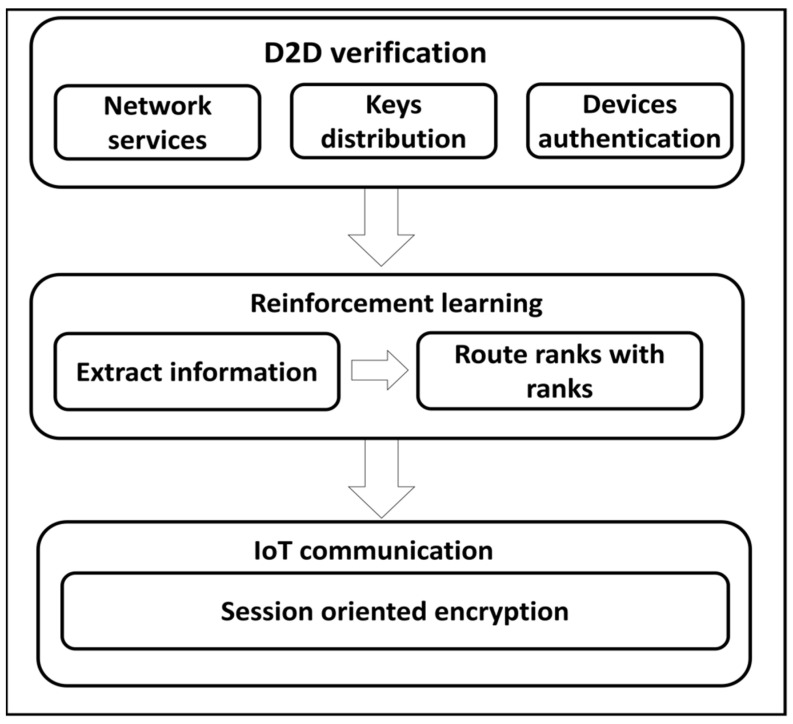
Development flow of the proposed algorithm.

The proposed algorithm is comprised of two stages. In the first stage, D2D authentication is performed, and afterward, using the machine learning approach the optimal forwarding tables are established. The forwarding tables are updated based on the network conditions, which decreases the overheads in determining the optimal routes. The second stage provides the trustworthiness forwarding in terms of privacy and integrity from the observing field to network applications. In this stage, the proposed algorithm ensures the accuracy of the collected data and eliminates the number of attacks from unknown devices. Additionally, the proposed algorithm imposes the lowest computing cost and data diverting for ensuring security between mobile devices, with nominal communication delays. Figure 1 illustrates the development flow of the proposed algorithm.

The contributions of the proposed algorithm are as follows:

- The first component is D2D authentication and key distribution. It consists of mobile devices and is associated with the inline gateway for obtaining the secret keys. Additionally, gateways are directly associated with the sink node for forwarding the network information to data centers.
- Reinforcement learning is executed in the second component by fetching the nodes’ statistics from the constructed forwarding tables along with information of packets’ reception. The forwarding tables are updated iteratively, thus the proposed algorithm converges the desired outcomes optimally. Using the machine learning technique, the proposed algorithm imposes lower routing overhead on the constraint devices and informs about the latest information to mobile nodes by exploring network parameters.
- The third component is secured IoT communication and accomplishing sustainable routing with the support of a D2D session-oriented system. It provides authentic and verifiable sessions between devices, gateways, and sink nodes with low-security costs.

### 3.1. D2D Authentication with Multi-Criteria Reinforcement Learning

In the beginning, the devices build a table containing their neighbor information, which is saved in their memories. We consider that the devices are mobile, and they advertise their current address when they are away from their home network. In the table, each device maintains the neighbors’ information, such as identity id, distance, $d_{i\;}$, residual energy, $e_{i\;}$, and radio coverage limit, $CR_{i\;}$, to next-level nodes. Moreover, as the devices are mobile, the proposed algorithm initiates the process of authentication using gateways, $w_{i\;},\;$by utilizing the session keys, $K_{s\;}$. All the nodes are required to distribute the tokens, $T_{k},\;$at the beginning of data forwarding, which consists of identity, timestamp, and positioning coordinates. Additionally, the token is encrypted using the obtained session key, from device x to device y. The session keys are temporary for a specific authentication process, and when the positioning coordinates of the devices are changed, the generated keys are revoked. Afterward, device x has to obtain a new session key from the proximity gateway for communication with its other peer devices. Each device generates a request with its id to the nearest gateway for mutual communication with a peer device. Upon receiving this information, the gateway constructs a record inside its table and generates a symmetric key sK for the peer devices over the secured channel. Later, both devices perform an encryption function, e, to securely transmit the data packets $m_{i\;}\;$as defined in Equations (1) and (2):

(1) $$w_{i\;}\to x:\;e_{sK_{\;}\;}\left(m_{i\;}\right)\;+d^{\prime }$$

(2) $$w_{i\;}\to y:\;e_{sK_{\;}\;}\left(m_{i\;}\right)\;+d^{\prime }$$

where $d^{\prime }$ shows the digital signatures. On the other hand, the devices first verify the validity of the encryption blocks using digital signature, and afterward, the peer nodes perform a decryption function to recover the data packets. In the proposed algorithm, each device updates the information in the constructed table and makes an entry of the authorized device as well. In case any device is found faulty, then its entry is removed from the table by the source device.

Most of the solutions [31,32] utilize multiple parameters for data aggregation and route the data in the network system. The proposed algorithm uses the concept of multi-criteria evaluation for data aggregation and optimizing the learning procedure in terms of constraint resources. The learning procedure also makes use of radio coverage, nodes’ mobility, and link cost to attain an energy-efficient and stable end-to-end communication system. In the proposed algorithm, each node obtains the information of the neighbor and utilizes the reinforcement learning technique for optimizing the intelligence process with nominal resources’ consumption. The source node initiates the process for the selection of the next-hop based on the highest rank. This route rank, R(i), denotes the most optimal neighbor, i, for decreasing the communication delay, energy consumption, and data disturbance, as defined in Equation (3):

(3) R(i)=rei +(1si )+CRi +1/lcosti,j 

where $re_{i\;}$ is residual energy, $s_{i\;}$is speed, $CR_{i}\;$is radio coverage, and $lcost_{i\;}$ denotes the link cost from node i to node j. $lcost_{i,j\;}$ is the integration of packet reception ratio, PRR, and average delay time, $ave_{dtime\;}$. To compute this, the source node distributes n number of probes’ packets in a fixed time interval, t, and as a result, the neighboring node j determines the value of $lcost_{i,j\;}$ for node i, as defined in Equations (4) and (5):

(4) lcosti =(PRR(i,j) +1avedtime )+1/derr 

(5) avedtime =(pn −pi )t

where $p_{n\;}$and $p_{i\;}$ denote the reception time for the first and last probe packets, t is the given time interval, and $d_{err\;}$is the data error, used to measure the number of retransmissions.

**Figure 2:**
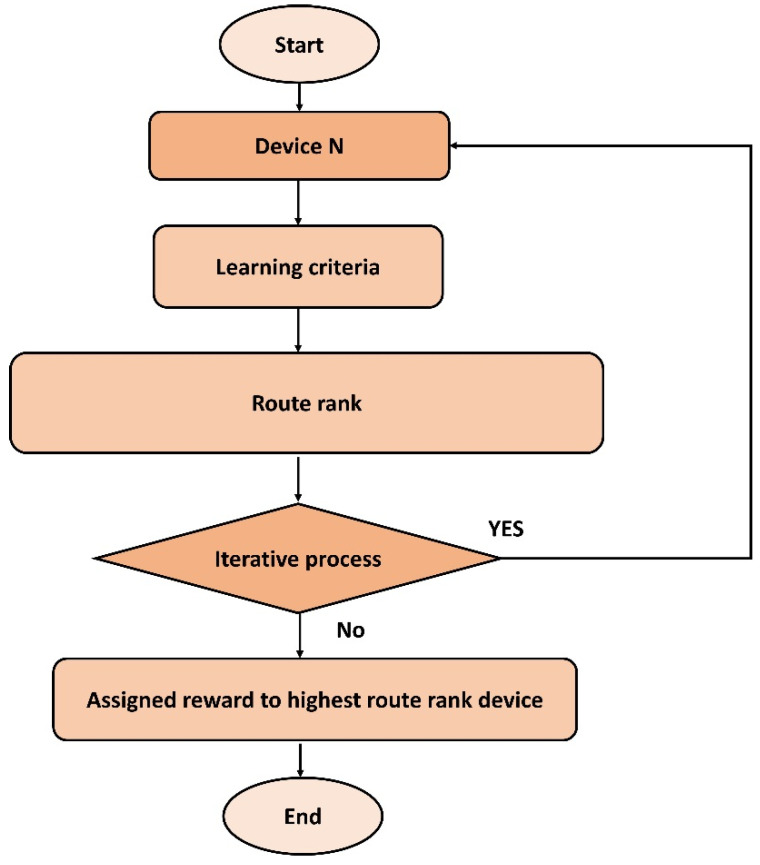
Route rank using reinforcement learning.

**Figure 3:**
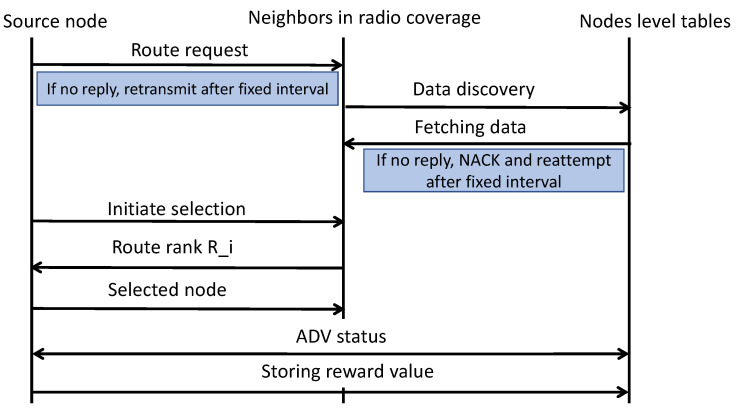
Next-hop selection procedure.

The proposed algorithm utilizes reinforcement learning [33] for computing and selecting the routing states using network conditions and experiences. The reinforcement algorithm is comprised of agents, states, S, and a set of actions, A, per state. Using reinforcement learning, node i exploits the R(i) values and selects the next hop using energy, speed, radio coverage, and link cost metrics. On receiving the data, the next-hop performs the re-computation of the R(i) value and forwards the data through its selected routing states. This process is continued for each neighbor selection until network data are received at the sink node. Additionally, when device i needs to route the data at the time $t_{0}$, it performs a set of actions and selects the neighbor node based on the computed route rank. The value of route rank is dynamically changed by evaluating the network and nodes’ statistics. Later, the device i gains a reward, Rwd, and enters the next state, i.e., (S, a, Rwd). A node has only a single reward value at any time. If any node has no reward value at any moment, then it will not be allowed to participate in the routing phase. On entering into the next state, the device i updates its forwarding table by adding the value of the reward. Moreover, the preceding device retrieves the updated information of device i. This practice of reinforcement learning is exploited by the proposed algorithm for finding the most optimal routes for forwarding the IoT data towards the sink node. At the end of the learning period, the entries of forwarding tables are converged to a numeric value that indicates the optimal route from the source device to the sink node. Converged forwarding tables with computation of route rank not only decreases the unnecessary data diverting but also increases the packets reception ratio over the communication channels in the existence of malicious nodes. Figure 2 illustrates the flow of reinforcement learning by exploiting the computed route rank. It uses the multi-criteria of the nodes to determine its rank value and accordingly assign the reward. Based on the updated forwarding tables and reward values the proposed algorithm offers convergence results and increases the route lifetime in terms of energy, speed, and link cost. The convergence levels depend on the number of iterations until end-to-end routes are established with the efficient distribution of constraint resources. Figure 3 shows the message flow for the selection of the next-hop between the source node and its neighbors. The source node floods the route request packet in its radio coverage and identifies the nearest neighbors. In a case when no reply has been received, then it resends the request packet. Once it has found the list of neighbors, then the process of data discovery is initiated, utilizing the node-level table to fetch the statistics. Based on the fetched data, the proposed algorithm computes the route rank using a multi-criteria process and the assigned reward value by exploiting reinforcement learning. Thus, selected nodes advertise their status for the connection in the routing phase, and sensors’ data is forwarded to the sink node.

The format of the node-level table is presented in Table 2.

### 3.2. Secured Data Transmission Using a Secured Session-Oriented Scheme

The proposed algorithm offers secure IoT-enabled smart data routing by utilizing the interaction of session keys between devices, gateways, and the sink node. This process is comprised of two levels. In the first level, the devices and gateways exchange their session keys and obtain the cipher information over the insecure channel. In the second level, the session keys are shared among the gateway and the sink node. Furthermore, session keys have an expiration time and are revoked after the completion of this time. However, we consider that the devices are mobile, so it might be a case that the device moves to another communication range, thus the session key is also revoked, and it sends a new request to the nearest gateway for providing the new session key and executes the authentication process. The session keys are encrypted using the public key. Let us consider that ${\left(ks_{i}\right)}^{n}$ denotes the set of session keys. Then, data encryption, E, from the mobile network device i to the gateway j can be obtained as shown in Equation (6). Before this, device i to the gateway j performs an authentication function to validate the session key, as defined in Equation (6):

(6) $$i\to j:E\left(ks_{i},\left[\;n_{i},\;t_{i}\;\right]\right)$$

where $t_{i}$ is a timestamp and $n_{i}$ is a nonce, also known as a random number. It is encrypted using the symmetric key of mobile device i. On receiving the encrypted session key, the gateway j includes its nonce, $n_{j}$, along with the timestamp, $t_{j}$, and sends back the confirmation message, as defined in Equation (7):

(7) $$j\to i:E\left(ks_{i},\left[\;n_{j},\;t_{j}\right]\right)$$

Accordingly, both devices on the network authenticate themselves, and now the network messages, $m_{i}$, can be ciphered using the encryption function, as provided in Equation (8):

(8) $$i\to j:\;xor\;(m_{i},ks_{i})\;$$

Finally, when data are received by gateways, they establish separate sessions with sink nodes using Equations (6) and (7). Afterward, the device data, M, are forwarded to the sink node, sink, including the digital signature, MAC, of the gateway with its private key, $R_{j}$, and ciphered data, $E\left[m_{i},ks_{i}\right]$, as shown in Equation (9):

(9) $$M\left(j,\;sink\right)=MAC(R_{j},E\left[m_{i},ks_{i}\right])$$

**Figure 4:**
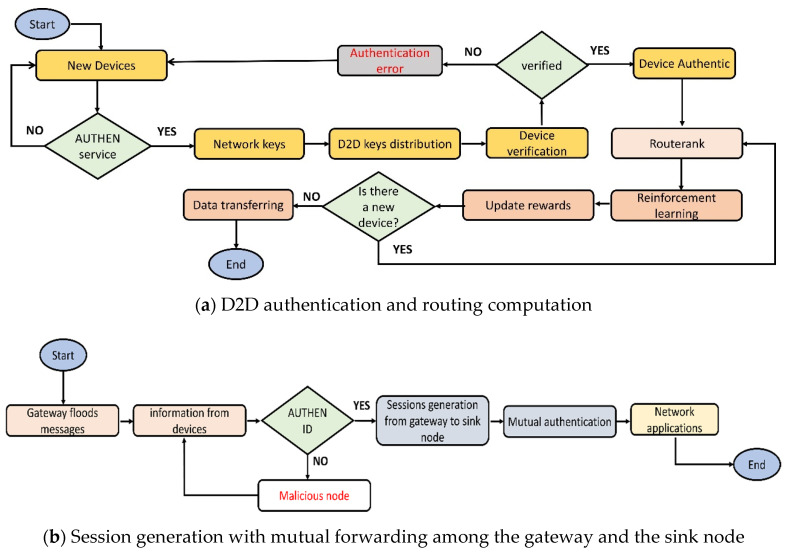
Flowchart of the proposed algorithm.

Figure 4a,b describes the flowcharts of the proposed algorithm. Initially, the network services and mobile devices gather the network data from the smart environment. Network keys are generated for D2D authentications, and after their verification, they can be a part of the routing. The proposed algorithm determines the value of route rank based on the multi-criteria and updates the nodes’ tables. Afterward, it utilizes reinforcement learning to assign rewards for the nodes. These rewards significantly improve the training process for the devices to extract the optimal neighbors from the set of choices, and accordingly, offer energy-efficient, least error rate delivery paths. Moreover, the proposed algorithm also secures the sessions among the gateway and the sink node for data transfer. Both the gateway and the sink node established secure sessions for their direct communication and are valid for a fixed time interval. After the mutual authentication, the gateways interact with the sink node for forwarding the network data with nominal communication costs.

**Figure 5:**
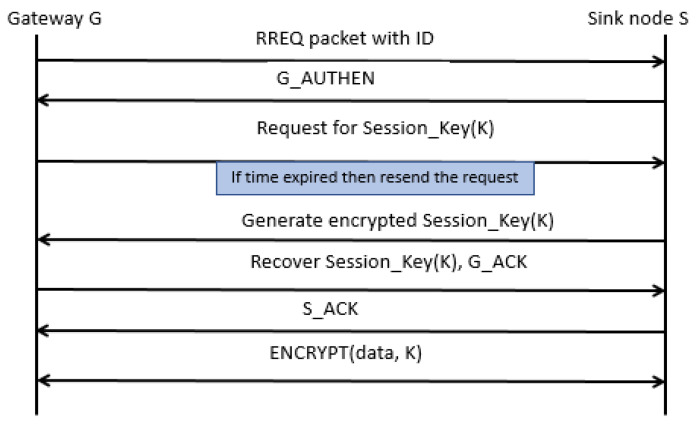
Message flow between the gateway and the sink node.

Figure 5 shows the flow of messages between the gateway and sink node for the establishment of a secure session with encrypted data transfer. In the beginning, the gateway device transmits the route request packet along with its *id* towards the sink node. Upon successful verification, the sink node acknowledges it, and later the gateway device requests the session key. If the time expires, the gateway device resends the request for the session key. Once the sink node receives the request for the session key, it generates the key and sends it towards the gateway device in encrypted form. The gateway device decrypts it and sends an acknowledgment message to the sink node that it has received the session key. The sink node confirms the acknowledgment message and afterward, both devices use the same session key for data encryption and decryption.

Algorithm 1 explains the pseudocode for the proposed work. It has two main components: one for the authentication of mobile devices with the reinforcement learning technique to assign the rewards, and the other for session-oriented data encryption from mobile sensors towards the sink node. After the successful verification of mobile sensors, the proposed algorithm evaluates the route rank for the neighbors using multiple parameters, along with the link cost. Accordingly, the neighbor with the highest route rank is assigned a reward value and selected as a forwarder. Moreover, the proposed algorithm also established a secure session from mobile sensors towards the sink node using gateway services. In this case, only that node is allowed to send the route request to the sink node that has a valid session key. The secure session key is utilized by both the mobile sensor and the sink node for data encryption and decryption, respectively.
**Algorithm 1:** Multi-criteria learning algorithm with secured devices.Input: SN: Sensor nodes RREQ: Route requestID: IdentityK: Session keyS: Sink nodeG: Gateway nodesOutput: Authentic devices, Dynamic routes, R, Secure transmission, Sec1. **for** i = 1:N2.  initiate Authen_service3.    if Authen_service = True4.     call keys_gen_process 5.    else6.     node is declared as faulty7. **end for**8.  **for** j = 1:G9.    **if** dist(j) closest to S10.    mutual_authen service 11.    encrypt(data, K)12.    **else** 13.     execute keys_gen_process 14.     mutual_authen service 15.     e = encrypt(data, K)16.    **end if**17. **end for** 18. **If** destination == S 19.     Recover K 20.     decrypt(e, K) 21. **end if**

## 4. Simulation Setup

This section presents the simulation configuration to evaluate the performance of the proposed algorithm. We experimented with the proposed algorithm, CTEER [25], and QL-MAC [27] solutions in terms of energy consumption, packet delivery ratio, packet disturbance, data latency, and computational complexity. The experiments were performed under varying rounds and the varying number of nodes using NS-3. Initially, nodes have homogeneous energy levels of 5 joules. The transmission range was set to 10 m. We deployed varying sensor nodes in the field of 300 × 300 m with a static sink. Sensor nodes are mobile with an installed GPS. Additionally, we assumed the number of malicious nodes to be 10. The data traffic between connected devices is a type of Constant Bit Rate (CBR). We assumed the energy model as discussed in [34,35]. Equations (10) and (11) define the energy consumption by exploiting the transmitted and received data bits:

(10) $$E_{tx}\left(k,d\right)=\{\begin{array}{c} E_{elect}*k+k*E_{fs}*d^{2}\;if\;d<d_{0}\\ E_{elect}*k+k*E_{amp}*d^{4}\;if\;d\;\geq \;d_{0} \end{array}$$

(11) $$E_{rx}\left(k\right)=E_{elect}*k$$

where $E_{tx}$ and $E_{rx}$ are the transmitting and receiving energy, k is data bits, d is the distance among sensor nodes, $E_{elect}$ is the amount of consumed energy per data bit, and the energy of the transmitting amplifier is denoted by $E_{fs}$. Table 3 illustrates the parameters for simulation configuration.

### Results and Discussion

In this section, we first evaluate the security test of our proposed algorithm against different possible attacks. In the proposed algorithm, the D2D communication is based on the authenticity and verification of devices. Once on the communication channels, the devices are verified, and then they generate session keys. Using the sessional keys, the device initiates the sharing of data over secure links. It might be possible that the session keys are compromised, so the proposed algorithm utilizes the encrypted procedure to forward the security keys. Additionally, session keys are automatically revoked, and each node has to send a new request to the gateway for issuing the session key. In our proposed algorithm, the devices are assumed as mobile, so when any node shifts to other coverage limits, then the obtained session key will not work and it generates a request packet to the new gateway for mutual association and further authentication. Table 4 shows the general network attacks and the procedures of the proposed algorithm used to avoid them.

**Figure 6:**
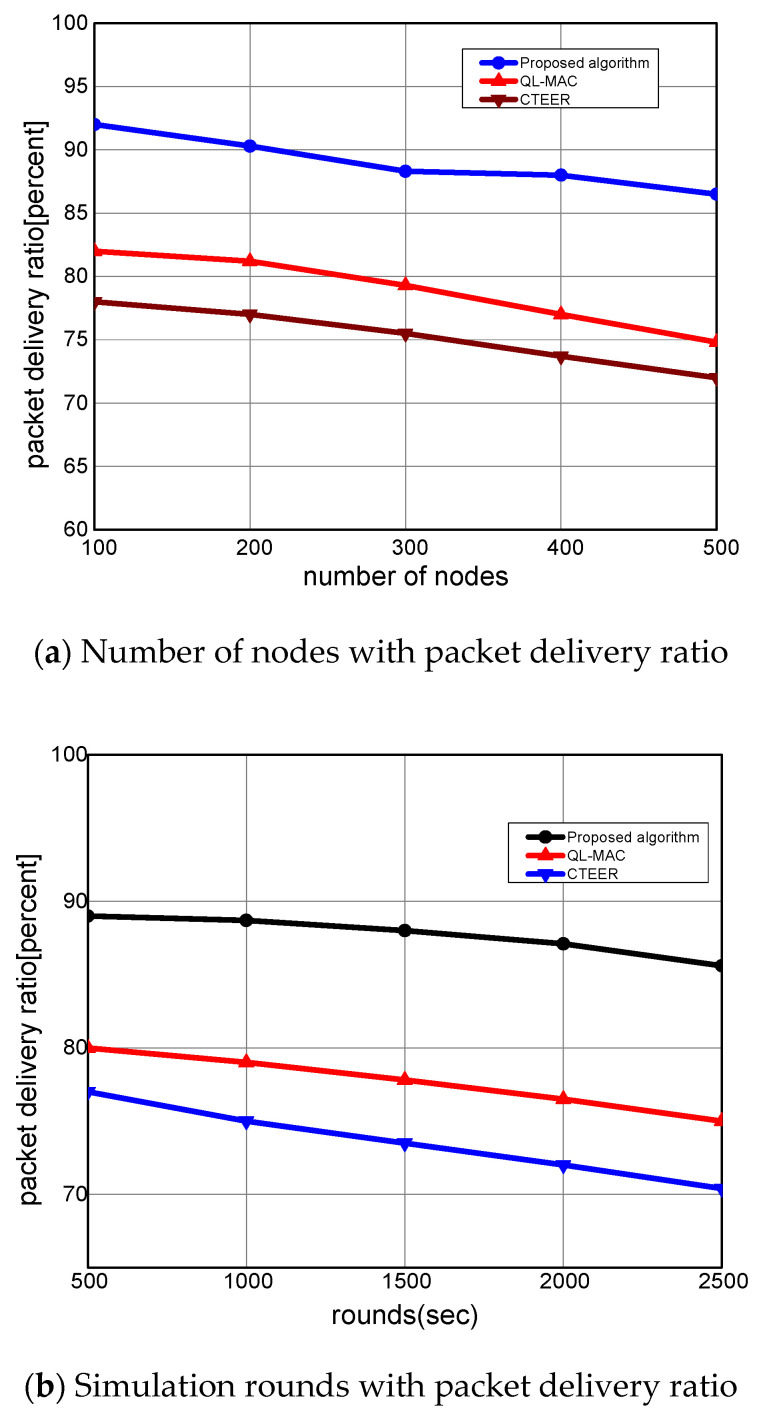
The performance evaluation of the proposed algorithm compared to QL-MAC and CTEER for packet delivery ratio.

In Figure 6a,b, the performance evaluation of the proposed algorithm is evaluated with other solutions for the packet delivery ratio. It can be computed as the ratio of the number of delivered packets to the total number of transmitted packets from the source node to the destination node. It is seen that with a varying number of nodes and rounds, the proposed algorithm increases the packet delivery ratio by an average of 18% and 17%. This is because the proposed algorithm utilizes the route rank function to estimate the aggregate condition of the devices. Unlike QL-MAC and CTEER solutions, the proposed algorithm periodically judges the situation of the mobile devices in terms of speed, coverage ratio, and packets information, and supports robust IoT-based routing development. Accordingly, it offers the most reliable neighbors for the selection of routing states. Moreover, the proposed algorithm utilizes the position of mobile sensors to balance the load among devices, thus ultimately increasing the delivery performance towards the sink node. Additionally, the sensing range is exploited by the route rank function to incorporate the high node density option in routing decisions. The proposed algorithm makes use of multi-hop mode for forwarding the network data rather than single-hop mode, and gateways perform the intermediate roles among mobile sensors and the sink node. Such an approach efficiently exploits the coverage option and robust connectivity among deployed devices and network applications.

**Figure 7:**
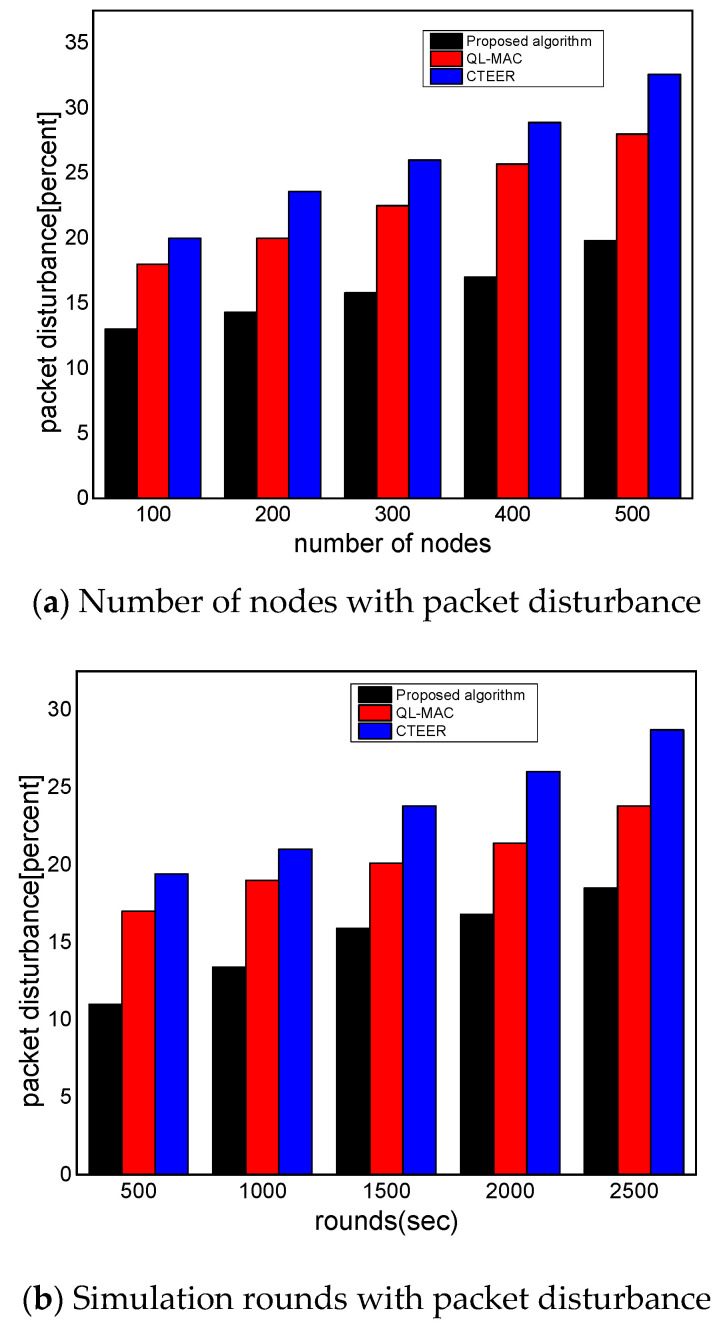
The performance evaluation of the proposed algorithm compared to QL-MAC and CTEER for packet disturbance.

Figure 7a,b illustrates the performance evaluation of the proposed algorithm for packet disturbance with existing solutions. It can be determined as a ratio of the number of packets lost to the data packets transmitted in a communication system. Compared to other work, the proposed algorithm improves the packet disturbance ratio by an average of 34% and 28% under a varying number of nodes and rounds. This is because the proposed algorithm does not overlook the constraint resource of the nodes and uniformly distributes the communication load among devices using reinforcement learning. Unlike QL-MAC and CTEER, the proposed algorithm utilizes network conditions in terms of multiple criteria and balances the data distribution on transmission links with the evaluation of the link cost. The proposed algorithm utilizes the PRR and response time factors in determining the optimal neighbors from the set of nodes. Additionally, with better utilization of the energy consumption of data forwarders, the proposed algorithm increases the strength of routes and prolongs the stability of the transmission system. Moreover, the D2D multi-criteria reinforcement learning-based routing decision avoids the chance of selecting faulty and overloaded links for the forwarding of IoT data. Based on the reward value, the proposed algorithm increases the efficiency for learning and offers a stable routing performance. Unlike QL-MAC and CTEER, the authentication and verification process of the proposed algorithm offers trustworthy communication among devices and supports improved packets’ distribution over the links.

**Figure 8:**
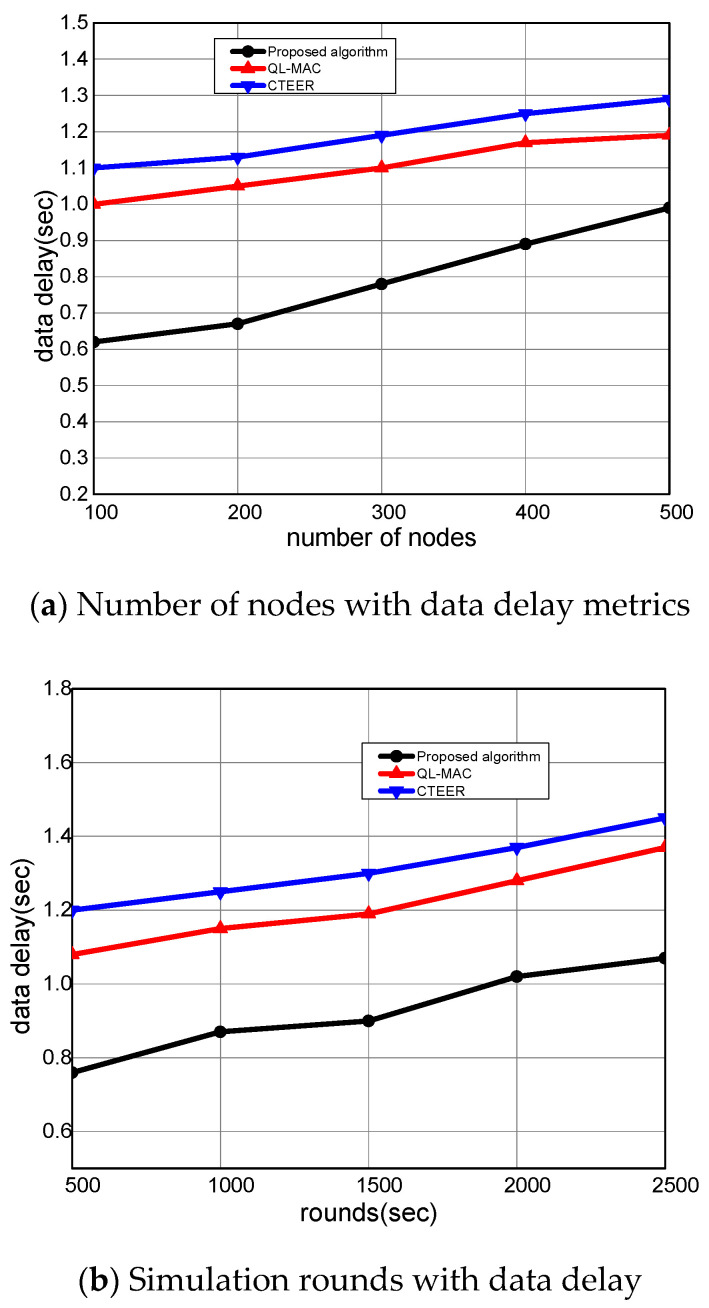
The performance evaluation of the proposed algorithm compared to QL-MAC and CTEER for data delay.

Figure 8a,b illustrates the experimental results of the proposed algorithm compared to the existing solution. It measures the round-trip time of forwarding the network data towards its destination in the communication system. It was observed that the proposed algorithm significantly decreased the data delay by an average of 20% and 23% for varying nodes and rounds. The proposed algorithm utilizes the concept of mobile sensors that rapidly shift their positions for the observation and forwarding of network data. In addition, the multi-criteria parameters in the forwarding scheme explicitly achieve optimal performance for constraint devices. Unlike most of the other proposed reinforcement learning schemes, the proposed algorithm assigns the appropriate rewards to nodes, decreasing the response time and data delay for smart mobile devices. Moreover, it uses the error rate metrics in determining the loss ratio, and thus only optimal neighbors whose link cost is not congested are chosen for data routing. The D2D direct authentication and verification in the routing of data packets also decrease the involvement of unauthorized nodes. Such an approach improves the transmission path, avoiding unnecessary delays and retransmissions. Unlike other solutions that impose overheads for securing the data forwarding and lead to a high delay rate, our proposed algorithm offers lightweight session keys based on secure routing, which explicitly minimizes the latency ratio on communication paths.

**Figure 9:**
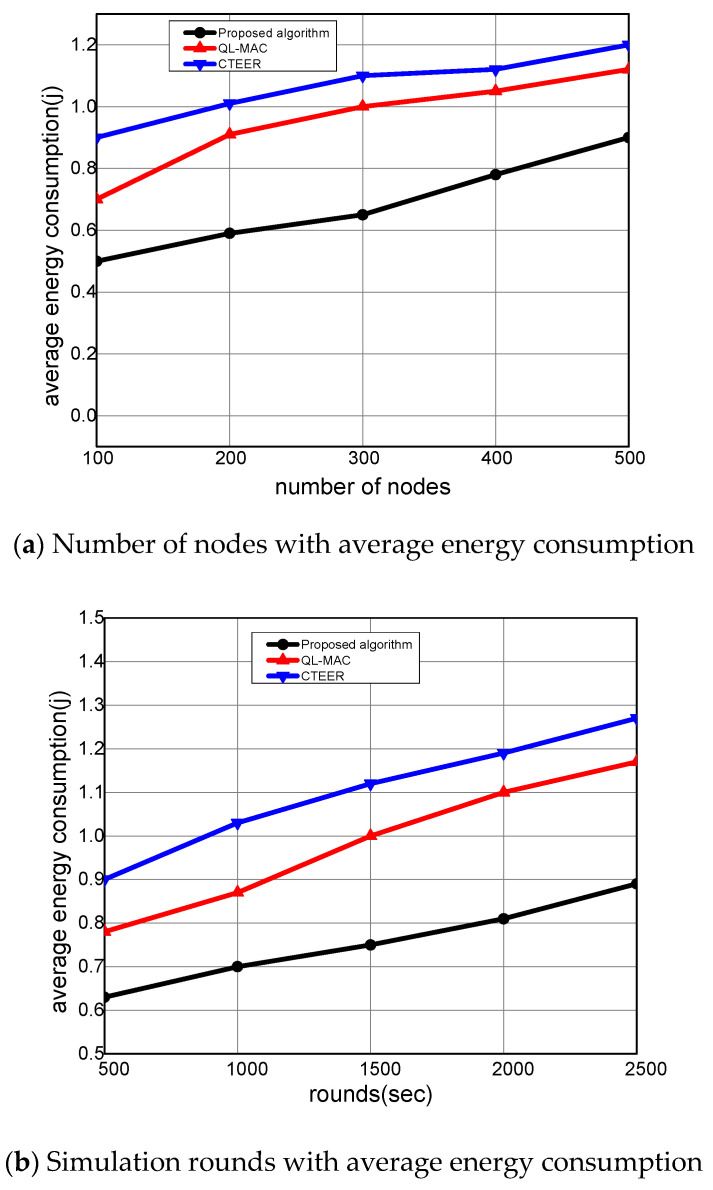
The performance evaluation of the proposed algorithm compared to QL-MAC and CTEER for average energy consumption.

In Figure 9a,b, the performance analysis of the proposed algorithm is evaluated compared to other solutions in terms of energy consumption. It is computed as a ratio of depleted energy to the total network energy in data sensing, receiving, and transmitting. It was found that the proposed algorithm minimized the energy consumption by an average of 22% and 27% under a varying number of nodes and rounds. This is because of the uniform load distribution among forwarders based on the machine learning technique. The reward value significantly trains the source node to fetch the previous information of the selected neighbors from its node-level table and optimize the performance for the constraint network. Moreover, it also decreases the extra energy consumption in sending the data from agricultural land using mobile sensors, which near uniformly balances the load on nodes. Additionally, only those nodes that fall into the coverage range exchange their information to proceed with the data routing. In case no node is found, then the next inline gateway device is assigned the responsibility of achieving the routing process. In all these processes, the proposed algorithm decreases the load on the mobile nodes and explicitly optimizes the consumption of energy resources.

**Figure 10:**
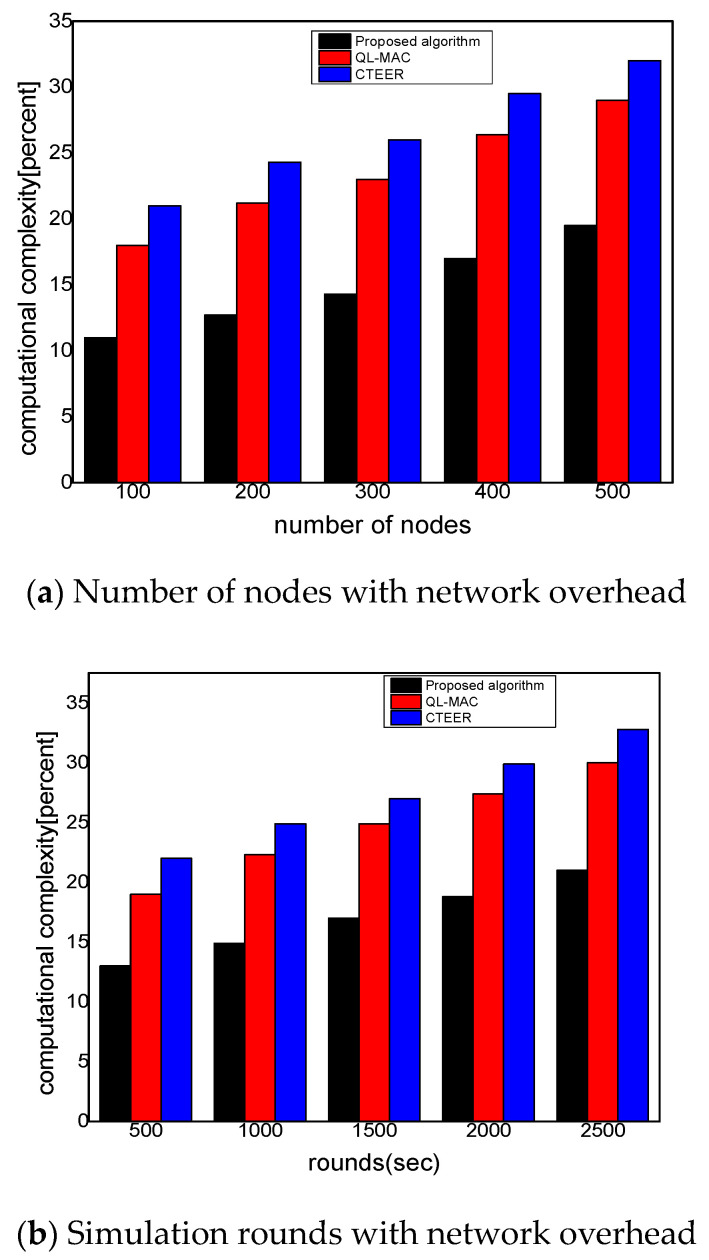
The performance evaluation of the proposed algorithm compared to QL-MAC and CTEER for computational complexity.

Figure 10a,b demonstrate the experimental results of the proposed algorithm compared to the existing solution for computational complexity. The results determined the number of processing overheads it takes to execute the proposed algorithm. It was seen that the proposed algorithm minimized the computational complexity by 36% and 39% for varying numbers of nodes and rounds. In computing the computational time, the proposed algorithm measures the number of route requests and route response packets, especially in the presence of malicious nodes. Moreover, it also considers the number of retransmissions in computing the computational time of the proposed algorithm. Based on the security function, the proposed algorithm efficiently identified the false requester, which significantly decreases the ratio for a computational time as compared to other solutions. Furthermore, using the reinforcement learning technique, balanced the resources’ consumption among the nodes and decreased the communication complexity by minimizing the least distance towards the sink node. The gateways perform the role of local supervision and reduced the cost of D2D communication by utilizing the method of coverage limit. Unlike QL-MAC and CTEER, the proposed algorithm supports the authentication and verification phase for mobile sensors and avoids the chance of malicious nodes generating excessive false traffic. Additionally, such security methods of the proposed algorithm impose the least communication complexity on constraint devices in the presence of malicious nodes, with affordable data retransmissions. Moreover, the link cost function identifies the more appropriate trusted links by utilizing the information of packets’ reception and data error.

## 5. Conclusions

IoT technology and sensor networks are widely utilized for monitoring, data collection, and analysis of smart environments using the wireless communication system. However, due to the constraints of resources of the nodes, most of the solutions are unable to balance the routing load on the selected routes and incur rapid data losses in the presence of security attacks. In this paper, we presented a D2D multi-criteria reinforcement learning algorithm with secured IoT infrastructure for smart cities. It offers a more authentic and verified solution for directly connected devices and increases the trustworthiness of transmission. Using multi-criteria reinforcement learning, the proposed algorithm offers intelligent methods for sensing the coverage area and efficiently distributing the energy load between mobile devices. The proposed algorithm can be used for smart buildings to interconnect various operations and for security surveillance using mobile IoT devices and sensors technologies. Our proposed algorithm makes it possible to gather the real-time data from the smart building and timely transmit the data towards network applications for further analysis and appropriate actions.

However, the proposed algorithm still suffers from link disruption with the high exchange of control packets, and thus in the future, we aim to utilize the deep learning model and real-time dataset to train the network nodes and cope with network anomalies. Additionally, we would like to introduce the concept of multi-clouds in the proposed algorithm for high scalability and parallel processing.

## Figures and Tables

**Table 1:** Summary of related discussion.

Comparative Approaches	Pros and Cons
Existing solutions	Most of the existing solutions have proposed for efficient utilization of energy consumption with constraint devices and improved the performance of data delivery. However, it is noticed that some solutions can tackle mobile devices at the cost of frequent data lost and overloaded wireless channels. Although machine learning techniques are explored by different researchers for IoT networks; however, it was seen that they overlooked security threats such as privacy, integrity, and authentication for mobile devices. Such a solution affects the reliability of smart cities and compromised communication system in the presence of unknown machines.
Proposed D2D multi-criteria learning algorithm using secured sensors technologies	An algorithm is developed for smart cities using reinforcement learning techniques based on devices and packets’ reception information. It supports gathering real-time data by imposing security restrictions for mobile devices against malicious actions. Moreover, mobile devices are verified first, and afterward, they are allowed to be involved in the data-gathering phase. It also supports data encryption with a session-oriented function and leads to lightweight complexity for the mobile network.

**Table 2:** Node level information.

1 Byte	1 Byte	1 Byte	2 Bytes	1 Byte	2 Bytes	1 Byte
Identity, id	$\mathrm{Energy},\;e_{i\;}$	$\mathrm{Distance},\;d_{i\;}$	$\mathrm{Link}\;\cos t,\;lcost_{i\;}$	$\mathrm{Radio}\;\mathrm{Coverage},\;CR_{i\;}$	Route rank, R(i)	Reward, Rwd

**Table 3:** Simulation configuration.

Parameter	Value
Simulation area	300 × 300 m
Deployment	Random
Propagation Model	Two Ray Ground
Node speed	5 m/s
Pause time	20 s
Malicious nodes	10
Simulations	10
Regular nodes	100–500
Initial energy	5 j
Transmission range	10 m
MAC layer	IEEE 802.11 b
Mobility model	Random waypoint
Simulation rounds	500–2500 s
Data traffic	CBR

**Table 4:** Security attacks and their related procedures.

Security Attacks	Proposed Procedures
Device authentication	Unique ID Session keys
Session key security	Encryption
Verification	Decryption using symmetric key
Confidentiality	Ciphered data using the session-oriented encryption
Malicious nodes regenerate request packet for session key	ID and session key expire automatically
Storage overload	Distributed data chunks and diffusion
Connectivity loss	Reinforcement learning
Additional resources’ consumption	Computing route rank
Network load	Distributed forwarding
Data originality	MAC, Digital hashes

## Data Availability

All data are available in the manuscript.
